# Breastfeeding technique and associated factors among breastfeeding mothers in Harar city, Eastern Ethiopia

**DOI:** 10.1186/s13006-018-0147-z

**Published:** 2018-01-30

**Authors:** Getahun Tiruye, Firehiwot Mesfin, Biftu Geda, Kasiye Shiferaw

**Affiliations:** School of Nursing and Midwifery, College of Health and Medical Sciences, Haramaya University, Harar city, Ethiopia

**Keywords:** Positioning, Attachment, Suckling, Breastfeeding technique, Breastfeeding in Eastern Ethiopia

## Abstract

**Background:**

Ineffective breastfeeding technique is one of the factors contributing to mothers practicing non-exclusive breastfeeding. Inappropriate breastfeeding technique is the leading cause of nipple pain in Ethiopia, as in other countries. However, no studies have examined breastfeeding technique and associated factors in eastern Ethiopia. Therefore, this study was conducted with the aim of assessing breastfeeding technique and associated factors among breastfeeding mothers.

**Method:**

We conducted an institution based cross-sectional study in selected governmental health facilities of Harar city, Eastern, Ethiopia, from January to February 2017. Systematic random sampling technique was used to select 422 study participants. Data were collected using pretested observational checklist and interviewer administered questionnaires containing sociodemographic, maternal and infant characteristics. The variables, positioning, attachment and suckling, were used to assess the outcome variable of breastfeeding technique. Descriptive, bivariate and multivariate logistic regression analysis was done to identify independent predictors of BFT after controlling for confounding variables.

**Result:**

The proportion of mothers practicing an effective breastfeeding technique was 43.4% (179/412). Effective breastfeeding technique was 2.3 times more common among mothers with at least secondary school educational status compared to mothers with no formal education (Adjusted Odds Ratio [AOR] 2.3; 95% Confidence Interval [CI] 1.1, 3.9). The practice of effective breastfeeding technique was significantly associated with mothers who had immediate breastfeeding technique counseling after birth (AOR 1.7, 95% CI 1.1, 2.8) and at least two postnatal visits (AOR 5.9; 95% CI 2.1, 15.9) compared to one visit. Absence of breast problems and having previous breastfeeding experience were also associated with the likelihood of effective breastfeeding technique practice (AOR 4.0; 95% CI 1.4, 10.9) and (AOR 3.3; 95% CI 1.1, 10.7) respectively.

**Conclusion:**

The practice of effective breastfeeding technique was low. Effective breastfeeding technique practice was associated with higher educational status, previous information about breastfeeding technique, previous breastfeeding experience, absence of breast problems, receiving breastfeeding technique counseling immediately after birth and at least two postnatal visits. Therefore; health services should provide education about effective breastfeeding techniques and ensure postnatal care for all women, particularly primipara.

## Background

Breastfeeding technique is the composite of positioning attachment and suckling [1]. Positioning refers to the technique in which the infant is held in relation to the mother’s body and attachment refers to whether the infant has enough areola and breast tissue in the mouth [2]. Proper positioning of the mother, good attachment of the baby to the breast and effective suckling is a function of effective breastfeeding technique [3, 4]. Performing effective breastfeeding technique (BFT) has been shown to be important to establish breastfeeding, to ensure milk transfer and to prevent breastfeeding problems [5–11].

Although breastfeeding technique is a natural act or phenomenon, it is not an instinctual behavior and requires a learned skill [9–12]. Once the mother knows the steps of effective BFT, she can better prevent and cope with most breastfeeding problem that might occur [9]. Most difficulties can be avoided together if good attachment and positioning are achieved at the first and early feeds [3].

A strong correlation between the onset of sore nipples and practice of ineffective breastfeeding technique among nursing mothers has been described [13, 14]. Thus, it is one of the major contributor to cause early cessation of breastfeeding practice [15]. A small localized based study in India indicated that ineffective BFT was present in about 57% and 63% of children suffering from diarrhea and ARI respectively [16].

Ineffective BFT results in insufficient intake of breast milk and this will cause poor weight gain and stunting and the baby may also become difficult to feed. Poor positioning, attachment and suckling also leads to sharp reduction of exclusive breastfeeding practice and increase breastfeeding problem [1, 15, 17–20].

Poor BFT was the leading cause of cracked nipple among breastfeeding mothers [13, 14]. Nipple damage and mastitis were more common among mothers with poor positioning and attachment [21]. Furthermore, breastfeeding problems were 2.44 times more common among mothers who practice ineffective BFT compared to who had effective BFT practice [22].

Several studies have proposed various solutions: education and counseling of mothers about effective BFT, training of health care professional with regard to good positioning, attachment and effective suckling, and giving special attention to young, primipara and poorly educated women [21–24]. Moreover; integrated management and childhood illness (IMNCI) guideline in Ethiopia also recommend that maternal and child health care providers are expected to ensure education and counseling of mothers about effective BFT and discourage supplementary feeding before six months [1]. Results from interventional studies have indicated that training of health care providers and continuing education, combining discussion with use of educational materials and practical demonstrations for breastfeeding mothers about positioning and attachment resulted in significant improvements in practice of effective BFT [24–26].

Although many studies about breastfeeding practice have been conducted in Ethiopia, the issue of breastfeeding technique has been overlooked. Therefore; this study was conducted with the objective of assessing BFT and associated factors among breastfeeding mothers attending governmental health facilities in Harar city.

## Method

The study was conducted in Harar city, Eastern, Ethiopia which is located 525 km from the capital city Addis Ababa. The population of the city is estimated to be 240,000 and 53,383 were women in the reproductive age group. There are 6 hospitals (4 government & 2 private hospitals) and 8 health centers which provide delivery and immunization services. The total number of eligible population in the city for antenatal care delivery and postnatal care was around 7604 [27]. This cross-sectional study design was conducted in Harar city governmental health facilities from 27 January 2017 to 26 February 2017.

### Study participants

All breastfeeding mothers in governmental health facilities of Harar city were the source population. Two groups of mother infant pair were recruited from postnatal unit and expanded program of immunization (EPI) unit. The study recruited immediate postpartum mother whose neonate was alive and mother coming for postnatal care visit at postnatal unit, and mothers coming for infant immunization service at EPI unit.

Therefore, all breastfeeding mothers who were in EPI unit and postnatal care unit in selected governmental health institutions (HIs) were included as the study population. Mothers who were seriously sick and unable to breastfeed, and whose infants were actually ill and refused to feed, very preterm birth and neonate with cleft lip and cleft palate were excluded from the study.

### Sample size calculation

Single population proportion formula was used to determine the sample size for this study with the assumption of 95% confidence interval (CI), margin of error 5% and 51% prevalence of effective BFT [22]. Considering 10% non-response rate, the total sample size was determined to be 422 breastfeeding mothers.

Four health facilities were selected by lottery method, and proportional allocation was done to select the sample from postnatal care unit and EPI unit of selected health facilities. Finally, systematic random sampling technique was employed to select study participant from postnatal care unit and EPI unit.

### Data measures

The study was conducted by using observation checklist and interviewer administered questionnaire containing sociodemographic, maternal and infant characteristics. An observation checklist was used to assess mother and baby’s position, infant’s mouth attachment and effective suckling during breastfeeding using World Health Organization (WHO) B-R-E-A-S-T- Feed Observation form [28]. The questionnaires were developed and modified from relevant studies in order to address all the variables necessary to meet the objective of this study [11, 21, 23, 29]. Interviewer administered questionnaires were prepared first in English and then translated in to Amharic and Afan Oromo language as per the mother tongue of the participant and translated back to English for consistency by language experts. The checklist and questionnaires were pre-tested on the population of 5% of the sample size of this study in Dire Dawa Dilchora Hospital. Then, correction and modification of the tool was undertaken accordingly. The following arbitrary scoring and grading system was developed and adopted to grade positioning (mother and infant), infant’s mouth attachment and effective suckling during breastfeeding based on WHO criteria [28]. Each criterion was assigned 1 point (Table 1).

**Table 1:** Grading system for infant’s body position, mouth attachment and effective suckling during breastfeeding for study conducted in HIs of Harar city, Eastern Ethiopia, 2017

Correct baby position:		
• Baby body should be straight and slightly extended.		
• Baby body close to the mother’s body		
• Whole body supported.		
• Baby facing toward the mother’s breast.		
Criteria for grading baby position in relation to mother:	Grade	Score
• None of or only one out of four criteria have been fulfilled.	0–1	Poor
• Any two of the four criteria have been fulfilled.	2	Average
• All the four/three criteria for infant positioning is fulfilled by mother.	3–4	Good
Correctness of attachment:		
• More areola is visible above the baby’s top lip.		
• The baby’s mouth is wide open.		
• The baby’s lower lip is turned outwards.		
• The baby’s chin is touching or almost touching the breast.		
Criteria for grading of correct attachment:	Grade	Score
• None of or only one out of four criteria have been fulfilled.	0–1	Poor
• Any two of the four criteria have been fulfilled.	2	Average
• Any three or all the four criteria have been fulfilled.	3–4	Good
Correctness of effective suckling:		
• Slow sucks		
• Deep suckling		
• Sometimes pausing		
Criteria for grading of effective suckling:	Grade	Score
• None of or only one of the three criteria have been achieved.	0–1	Poor
• Any two or the three criteria have been achieved.	2–3	Good

### Operational definitions

#### Effective breastfeeding technique (BFT)

The combination of at least two criteria from positioning, three criteria from attachment and two criteria from suckling are fulfilled while mothers breastfeed their infants [22].

**Parity** - Indicates the number of pregnancies reaching viable gestation.

- Low birth weight - weight at birth of less than 2.5 kg.
- Normal birth weight – weight of neonate at birth above or equal to 2.5 kg but less than 4 kg.
- Macrosomia – weight of neonate at birth above 4 kg.
- Preterm birth – a neonate born before 37 completed weeks. But for this study it is operationalized as age at birth between 34 and 36 completed weeks of gestation.
- Term - a neonate born any time after 37 completed weeks of gestation and up until 42 completed weeks of gestation.
- Post-term - a neonate born any time after completion of the 42 weeks.

**Breast problem** – are problems if a woman has any of the following:

- Inverted nipple - a portion of or the entire nipple is buried below the plane of the areola and does not evert at all (grade III) [30];
- Engorgement – breasts are painfully overfull;
- Cracked nipple – damage to the integrity of the skin on the nipple;
- Mastitis – inflammatory condition of the breast, which may or may not be accompanied by infection [31].

### Data collection

Eight female bachelor science (BSc.) midwives and two supervisors who were not working in the assigned health facilities were trained by the investigators on how to collect data and observe the positions of the mother and baby, infant’s mouth attachment to breast and effective suckling by using video clips and hands on practice. In addition, they observed BFT with clients at nearby health facilities. Data collectors observed the breastfeeding process for five minutes and recorded the mother and infant’s positioning, attachment to the breast and effective suckling as per WHO B-R-E-A-S-T Feed observation form. It was done by asking the mother to put her infant to the breast, if the infant had not been fed in the previous hour. If the infant had been fed during the last one hour, then the mother was asked when the infant would have the next feed and the observation assessment was planned accordingly. The investigator and supervisor closely followed the data collection process throughout the data collection period. They checked the collected data for completeness each day and corrective measures were taken accordingly.

### Data processing and analysis

The data were first coded, double data entry were done by two data clerks and consistency of the entered data were cross checked by comparing the two separately entered data and cleaned using EpiData statistical software version 3.1. Entered data exported into statistical package for social science (SPSS) software version 21 for analysis. The three variables i.e. positioning, attachment and suckling were used to create a single variable of BFT [22]. The outcome variable BFT was classified as either effective BFT or ineffective BFT. Descriptive statistics of each variable was determined and the results were presented in texts, tables and graphs using summary measures such as percentages, mean and standard deviation. Hosmer-Lemeshow’s and Omnibus goodness-of-fit test was used to assess whether the necessary assumptions for the application of bivariate and multivariate logistic regression. In the Hosmer-Lemeshow test, the Pearson’s chi-square should not be significant but it should be significant in Omnibus test if the model said to be fitted. Bivariate logistic regression was carried out to identify the predictors associated with BFT. All variables with *p*-value of ≤0.25 in bivariate logistic regression were included in the multivariable model. In the multivariate analysis, standard enter techniques were fitted. Variables having *p* value ≤ 0.05 in the multivariate analysis were taken as significant predictors. Crude odd ratios (COR) and adjusted odds ratios (AOR) with their 95% confidence intervals (CI) were calculated.

## Results

### Sociodemographic characteristics of the participants

Of 422 study participants, 412 were observed for BFT and interviewed, giving a response rate of 97.6%. Interview and observation was done on a total of 194 (47.1%) breastfeeding mothers from postpartum ward or postnatal care unit and 218 (52.9%) breastfeeding mothers from EPI unit. About 44 % 181(43.9%) of mothers were in the age group of 20–25 years. The majority of the respondents (76.7%) were housewives, and the mean age of the study participants were 25. 8 years with SD ± 5.34 years (Table 2).

**Table 2:** Sociodemographic characteristics of the participants for the study conducted in HIs of Harar City, Eastern Ethiopia, 2017 (*n* = 412)

Variables		Frequency	Percentage
Residence	Urban	261	63.3%
	Rural	151	36.7%
Age	< 20	49	11.9%
	20–25	181	43.9%
	26–30	112	27.2%
	> 30	70	17%
Religion	Muslim	272	60.0%
	Orthodox	127	30.8%
	Protestant	13	3.2%
Ethnicity	Oromo	275	66.7%
	Amhara	92	22.3%
	Harari	16	3.9%
	Other^a^	29	7%
Marital status	Married	395	95.9%
	Single	9	2.2%
	Other^b^	8	1.9%
Respondents’ educational status	No formal education	157	38.1%
	Primary school	124	30.1%
	Secondary school & above	131	31.8%
Occupation	House wife	316	76.7%
	Government employee	44	10.7%
	Non-government	9	2.2%
	Private employee	15	3.6%
	Daily laborer	11	2.7%
	Other^c^	17	4.1%
Number of family members	< 5	338	82.0%
	≥ 5	74	18.0%

Other ^a^Gurage, Tigre, Somali and Wolaita. Other ^b^widowed, divorced, other ^c^merchant, student and farmer and *HIs* Health Institutions

### Maternal characteristics of the participants

Just over half the participants had received information about BFT (52.9%). Of the total participants, the majority (85%) did not think that breastfeeding is painful and 18.7% complained that BFT takes time. Regarding breastfeeding experience, 60% had previous breastfeeding experience; 9.3% had previous breastfeeding experience < 1 years, 56.3% and 34.4% had previous duration of breastfeeding experience 1 year to 2 years and above 2 years respectively. From 412 study participants, 35 (8.5%) were diagnosed with a breast problem at the time of data collection. Engorgement was the commonest breast problem, accounting for around 46%, followed by cracked nipple and inverted nipple (each 20%), followed by mastitis or other problems.

### Obstetrics and infant factors

Nearly two thirds (61.7%) of participants were multiparous; hospitals were the commonest place for delivery (87.4%) and only 2.2% gave birth at home (Table 3).

**Table 3:** Obstetric and infant characteristics of participants for the study conducted in HIs of Harar City, Eastern Ethiopia, 2017 (*n* = 412)

Variables		Frequency	Percentages
Parity	Primipara	158	38.3
	Multipara	254	61.7
Antenatal care	Yes	349	84.7
	No	63	15.3
Antenatal Counseling about BFT	Yes	149	42.7
	No	200	57.3
Place of delivery	Hospital	360	87.4
	Health center	43	10.4
	Home	9	2.2
Mode of delivery	Normal delivery	341	82.8
	C/S	71	17.2
Immediate BFT counseling after delivery	Yes	144	35
	No	268	65
Postnatal care	Yes	405	98.3
	No	7	1.7
Frequency of postnatal care visit	1	373	92.1
	≥ 2	32	7.9
GA of the infant	Preterm	46	11.2
	Term	352	85.4
	Post term	14	3.4
Birth weight	LBW	37	9.0
	NBW	335	81.3
	Macrosomia	31	7.7
Age of the infant	< 42 days	218	52.9
	≥42 days	181	43.9
Infant’s sex	Male	202	49
	Female	210	51

*GA* Gestational Age, *BFT* Breastfeeding Technique, *LBW* Low Birth Weight, *NBW* Normal Birth Weight and *HIs* Health Institutions

### Effective breastfeeding technique

**Fig. 1:**
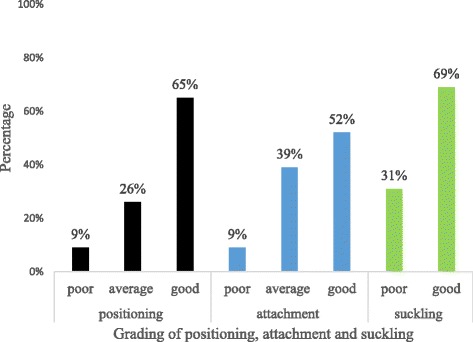
Proportion of positioning, attachment and suckling of infants during feeding in Harar City, Ethiopia, 2017

**Fig. 2:**
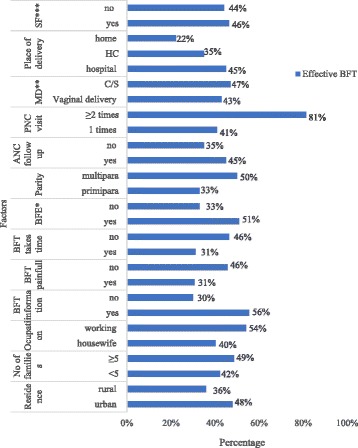
Effective breastfeeding technique based on sociodemographic, maternal and obstetric factors in Harar City, Ethiopia, 2017*.* BFE*: Breastfeeding experience, MD**: Mode of delivery, SF***: Supplementary feeding, C/S: Cesarean Section, BFT: Breastfeeding technique and HIs: Health Institutions

The proportion of effective BFT was 42.2% (82/194) in the postnatal unit and 44.5% (97/218) in the EPI unit. Good positioning (65.3%) and good suckling (69.2%) were observed during breastfeeding (Fig. 1). Effective BFT was more common among mothers who had at least two postnatal visits (81.3%), information about BFT (55.5%), working mothers (54.2%) and previous breastfeeding experience (50.6%) (Fig. 2).

### Factors affecting breastfeeding technique

Regression analysis was run in order to identify variables with significant association with the condition of BFT. In multivariable analysis model, there was a statistically significant positive association with respect to effective BFT among mothers who have educational status of secondary school and above (AOR 2.3; 95% CI 1.1, 3.9), those who had previous breastfeeding experience (AOR 3.3; 95% CI 1.1, 10.7) and absence of breast problems (AOR 4.0; 95% CI 1.4, 10.9). Practice of effective BFT was 1.9 times more common among mothers who had information about BFT (AOR 1.8; 95% CI 1.1, 3). Likewise, mothers who had at least two postnatal visits had increased odds of performing effective BFT practice compared to their counterparts (AOR 5.9; 95% CI 2.1, 15.9). Similarly, there was an association with practice of effective BFT among mother who had immediate counseling about BFT after birth (AOR 1.7; 95% CI 1.1, 2.8) (Table 4).

**Table 4:** Bivariate and multivariable analysis of factors associated with effective BFT among breastfeeding mothers in HIs of Harar City, Eastern Ethiopia, 2017 (*n* = 412)

Variables		Practice		Crude odd ratio (95%CI)	Adjusted odd ratio (95%CI)
		Effective Breastfeeding Technique (%)	Ineffective Breastfeeding Technique (%)		
Age of mother	< 19	28.6	71.4	1	1
	20–25	36.5	63.5	1.4 (.72, 2.8)	0.7 (0.3, 1.6)
	26–30	57.1	42.9	3.3 (1.6, 6.9)	1.3 (0.5, 3)
	> 30	50	50	2.5 (1.2, 5.4)	0.9 (0.4, 2.7)
Educational status	No formal education	36.3	63.7	1	1
	Primary	38.7	61.3	1.1 (0.7, 1.8)	1.3 (0.8, 2.2)
	2^ndary^ & above	56.5	43.5	2.3 (1.4, 3.7) *	2.3 (1.1, 3.9) *
Occupation	HW^a^	40.2	59.8	1	1
	Working	54.2	44.8	1.7 (1.1, 2.8)	0.8 (0.5, 1.5)
Previous information about BFT	Yes	55.5	44.5	2.9 (1.9, 4.3) *	1.9 (1.1, 3) *
	No	29.9	70.1	1	1
Breastfeeding experience	Yes	50.6	49.4	2.1 (1.4, 3.2) *	3.3 (1.1, 10.7) *
	No	32.7	67.3	1	1
Breast problem	Yes	14.3	85.7	1	1
	No	46.2	53.8	5.1 (1.9, 13.5) **	4.0 (1.4, 10.9) **
Parity	Primipara	32.9	67.1	1	1
	Multi para	50	50	2 (1.3, 3)	0.6 (0.2, 1.9)
Antenatal care follow up	yes	45	55	1.5 (0.8, 2.6)	1.3 (0.6, 2.4)
	No	34.9	65.1	1	1
Immediate counseling about BFT	Yes	59	41	2.6 (1.8, 4)*	1.7 (1.1, 2.8)*
	No	35.1	64.9	1	1
Frequency of postnatal care visit	1	41	59	1	1
	≥ 2	81.3	18.7	6.2 (2.5, 15.5)***	5.9 (2.1, 15.9)***

*, ** and ***: *P* value; ≤ 0.05, ≤ 0.01 and ≤0.001 both in the Crude and adjusted odds ratio respectively

*HW*^*a*^ Housewife, *BFT* Breastfeeding Technique and *HIs* Health Institutions

## Discussion

We found that the overall prevalence of effective BFT was 43.4%. The prevalance was lower than in a study done in west Denmark (52%) [22]. This discrepancy might be due to routine in service delivery of information and demonstration about effective BFT for breastfeeding mothers or it might be due to differences on awareness level of the mothers and educational status.

Our result showed that the proportion of effective BFT was nearly similar in the postnatal care unit (42.3%) and EPI unit (44.5%). This showed that the practice of effective BFT is not affected by being immediately after delivery or after a time, rather the awareness and educational level of mothers in particular and access to postnatal care services.

The educational status of the respondents had a significant impact on parctice of effective BFT. Mothers who had secondary school education and above were 2.3 times more likely to practice effective BFT compared to their counterparts. This was in accordance with a study done in Indian East Delhi, west Bengal Kolkata hospital, Saudi Heraa general hospital and Sri Lanka [11, 23, 32, 33]. This is due to the fact that more educated mothers had strong intention and willingness to breastfeed. This factor positively affected the ability of mothers to give more emphasis on instruction, guidance and support from maternal and child health care providers to have successful breastfeeding technique [34–36]. Moreover; this might be due to, educated mothers are more likely to acquire and observe BFT from their edacational carrier or which might be due to the fact that mothers of poor educational status take more time to adopt the correct breastfeeding technique.

The present study was able to demonstrate that mothers who had previous information about BFT was 1.9 times more likely to practice effective BFT compared to their counterparts. Likewise, lack of information about BFT in India, East Delhi had significantly influenced the practice of effective BFT [11]. The process of the association can be, mothers who had awareness are more eager to know about the importance of BFT and this made them how to apply the technique in a step wise practical fashion.

In this study, effective BFT was more commonly observed among mothers who had previous breastfeeding experience compared to mothers who had no prior experience. This was consistent with a study done in India, East Delhi and western Denmark [11, 22]. Possible explanations might be that they had acquired skills from practice or they might have access to support and guidance previously.

Breast problems like cracked nipples, mastitis and engorgement were significantly associated with prevalance of ineffective BFT. This was in congruent with studies held in Libya Benghazi hospital and Pakistan Khyber Teaching Hospital [21, 37]. Breast engorgement makes it more difficult for the newborn infant to latch-on correctly because of distension and edema of the nipple and areolar region [38]. Poor BFT can also lead to nipple damage and mastitis.

In our study, presence of routine provision of immidiate counseling and demonestration about effective BFT after delivery had significant impact on practice of effective BFT, which is consistent with several studies done in Bangladesh, Saudi Heraa general hospital, rural area of north India and rural area of Nagpur district [24, 32, 39, 40]. Moreover; having at least two postnatal visits two increased the likelihood of effective BFT (81.3%) compared to mothers who had only one postnatal visit (41%). This was true in Bangladesh among two groups of postpartum mothers, there were a significant positive association with practice of effective BFT among mothers who received both early and late postnatal care visit compared to only one visit by Community health worker [24]. This is likely to be due to the fact that psychological support for breastfeeding mothers through early counselling and hands-on support for achieving proper techniques, particularly position and attachment [33].

This study was the first of its kind to give an insight into the status of BFT in the study area. We used both an observation checklist and interview to assess BFT to ensure a methodologically sound study. However, study limitations include the lack of a validated WHO instrument and the possible Hawthorne effect [41].

## Conclusions

The study showed that proportion of effective BFT was low. The practice of effective BFT was mainly affected by educational status, presence of previous breastfeeding experience, mothers access to educational counseling immediately after delivery about BFT and prior information about BFT. Moreover; there were a significant positive association with effective BFT among mothers who have postnatal care visit ≥2 and those not identified with breast problem during data collection. Harari Regional Educational Bureau in collaboration with Ministry of Health should advocate women’s education and strengthen existing positive activities. All maternal and child health care provider in Harari Regional State Health facilities expected to work on information education and communication and demonstration for breastfeeding mothers and should consider it as their routine activities. Maternal, neonatal and child health care providers and, health extension workers expected to ensure postnatal care utilization. On top of that, prime attention, guidance and support should be given for mothers, who are novice to breastfeeding and have breast problem.
